# Developing a Vulnerability Mapping Methodology: Applying the Water-Associated Disease Index to Dengue in Malaysia

**DOI:** 10.1371/journal.pone.0063584

**Published:** 2013-05-08

**Authors:** Sarah K. Dickin, Corinne J. Schuster-Wallace, Susan J. Elliott

**Affiliations:** 1 School of Geography and Earth Sciences, McMaster University, Hamilton, Ontario, Canada; 2 United Nations University Institute for Water Environment and Health, Hamilton, Ontario, Canada; 3 Faculty of Applied Health Sciences, University of Waterloo, Waterloo, Canada; Northeastern University, United States of America

## Abstract

The Water-associated Disease Index (WADI) was developed to identify and visualize vulnerability to different water-associated diseases by integrating a range of social and biophysical determinants in map format. In this study vulnerability is used to encompass conditions of exposure, susceptibility, and differential coping capacity to a water-associated health hazard. By assessing these conditions, the tool is designed to provide stakeholders with an integrated and long-term understanding of subnational vulnerabilities to water-associated disease and contribute to intervention strategies to reduce the burden of illness. The objective of this paper is to describe and validate the WADI tool by applying it to dengue. A systemic ecohealth framework that considers links between people, the environment and health was applied to identify secondary datasets, populating the index with components including climate conditions, land cover, education status and water use practices. Data were aggregated to create composite indicators of exposure and of susceptibility in a Geographic Information System (GIS). These indicators were weighted by their contribution to dengue vulnerability, and the output consisted of an overall index visualized in map format. The WADI was validated in this Malaysia case study, demonstrating a significant association with dengue rates at a sub-national level, and illustrating a range of factors that drive vulnerability to the disease within the country. The index output indicated high vulnerability to dengue in urban areas, especially in the capital Kuala Lumpur and surrounding region. However, in other regions, vulnerability to dengue varied throughout the year due to the influence of seasonal climate conditions, such as monsoon patterns. The WADI tool complements early warning models for water-associated disease by providing upstream information for planning prevention and control approaches, which increasingly require a comprehensive and geographically broad understanding of vulnerability for implementation.

## Introduction

Water-associated diseases account for approximately 10% of the global disease burden, representing a significant source of morbidity and mortality worldwide [1]. These infections are spread by waterborne agents (eg. *E.coli* O157:H7, *Vibrio cholerae* O139*)*, vectors carrying viruses and parasites (eg. dengue, malaria), and water contact (eg. schistosomiasis). Re-emerging and newly emerging water-associated diseases present a further threat. For instance, Latin America has seen a re-emergence of dengue fever, despite previous eradication of the mosquito vector [2], [3]. Large outbreaks of chikungunya, an emerging virus also carried by *Aedes aegypti,* the primary dengue vector, have recently been reported in Asia and Africa [4], [5]. The recent introduction of West Nile virus in North-America was characterized by rapid dispersal of the virus and the largest outbreak of human encephalitis encountered in that region to date [6]. These illnesses have a global impact that is likely to be exacerbated by global environmental change and it is clear that the burden is disproportionately borne by the most vulnerable: the poor, women and children, and populations in low- and middle-income countries [7], [8]. Due to time spent supplying their households with food and water and caring for the sick, these groups often have less capacity to invest in resilience building activities.

While there is increasing knowledge of the linkages between water-associated disease and global environmental change, expansion of emerging infections demonstrates gaps in understanding of the complexity of these systems and their relationship to human health [9], [10]. For example, diseases such as schistosomiasis have resurged in areas where large-scale drug administration efforts were not sustained after initial targets were met [11]–[13]. Integrated control approaches are critical to addressing water-associated diseases impacted by a range of environmental and social factors, and there is a growing need for tools to assess vulnerability at the water-health nexus.

### Assessing Vulnerability to Water-associated Disease

Vulnerability is described as the condition of a system, or a propensity to be adversely affected [14], [15]. It encompasses exposure to harmful environmental or social stresses, susceptibility to these stresses, and the capacity to cope or adapt [16], often within the context of a particular hazard [17]. In this conceptualization of vulnerability, susceptibility represents the existing social, cultural, and economic conditions that render a population sensitive to impacts from a water-associated disease, while exposure represents conditions conducive to the presence and transmission of a water-associated pathogen within the environment.

Vulnerability assessment is an approach used to describe the potential for harm from a diverse range of hazards at local, regional, national or global scales [18]. A range of biophysical, social, economic, or cultural factors may be used as indicators of vulnerability. Natural disasters and climate change hazards have been a particular focus of vulnerability assessment, examining a range of environmental impacts such as flooding, wildfires and loss of ecosystem services [19], [20]. Poverty, livelihoods and heat related illnesses have also been the subject of vulnerability research [21]–[23].

While tools are being developed to assess vulnerability to hazards such as climate change, health impacts are generally evaluated through a risk framework. In the case of dengue, a range of disease risk models have been developed, including simulations of dengue transmission in a human population, models focusing on climate driving factors such as temperature and humidity, and ecological niche modeling of mosquito vector populations (e.g., [24]–[27]). Risk models are advantageous because they can inform early warning systems which attempt to predict outbreaks, but generally focus on a limited range of variables [28]. Researchers have argued for an expanded transdisciplinary approach to combat water-associated disease, as a large number of factors such as climate patterns, land use and socioeconomic determinants are often examined separately and with limited successes [10], [29]–[31]. For instance, using only the *Aedes* mosquito index based on larval surveys has been ineffective in predicting dengue incidence in many regions including Malaysia, Taiwan and Trinidad [24], [32], [33]. However, a review of risk models and early warning systems for dengue found that limited studies have been able to collect enough epidemiological, spatial and temporal data to examine the correlation between these factors [28]; emphasizing the need for pragmatic tools which are not geographically constrained due to data availability.

Vulnerability assessment offers a novel way to conceptualize the complex web of factors and interactions mediating the water-associated disease burden, by focusing less on the likelihood of the hazard occurring, and instead on analyzing a wide range of factors that impact exposure, susceptibility, and ability to cope and recover from a disease. While not a predictive approach, vulnerability analysis can synthesize social and biophysical information such as climate model thresholds and social determinants of disease to describe differential drivers of vulnerability, such as conditions which may or may not lead to an increased burden of illness in areas where a population is exposed.

This perspective can provide key information for decision-makers to create long-term health promoting interventions upstream of predictive early warning models [34], [35].

Thus, there is a need to develop and validate novel mapping and assessment tools to better target vulnerable areas in a cost-effective manner [30], [36]. To meet this need, a vulnerability mapping methodology was developed in the form of a Water-Associated Disease Index (WADI), integrating a range of social and biophysical components. The WADI can be constructed and applied by end-users in data-rich or data-poor regions to assess vulnerability to individual water-associated diseases, especially in the face of global environmental change.

The objective of this paper is to apply and validate the WADI vulnerability mapping approach in the context of dengue fever in peninsular Malaysia. This case study was carried out in partnership with the United Nations University International Institute for Global Health in response to a Strategic Plan on Dengue Prevention and Control to reduce dengue cases by 10% yearly in Malaysia and the Southeast Asian region through innovations to prevent and control the disease. Malaysia is one of many tropical countries where dengue is endemic and a major public health concern; the number of yearly reported cases remains very high. Approximately 47000, 41000, 46000 cases were reported in 2008, 2009, 2010 respectively [37].

### Use of Index Approaches

Vulnerability assessments often employ indicators to simplify and distil complex, real-world information into a format that is relevant and useful for decision-making. While indices provide an essential suite of tools at the science–policy interface, they have received a range of criticism for the way in which information is reduced into a single output [38]–[40]. For instance, approaches for selecting and weighting indicators to create meaningful composite indices have been subject to debate. One of the most common approaches employs equal weighted averages (eg. [41]–[43]), which ensures transparency and straightforward construction of an index, but has been criticized for assignment of implicit equal weights [44]. Other methods to weigh indicators include multivariate analyses and stakeholder or expert rankings; however no approach is without limitations due to the complexity of the systems represented [17], [45], [46].

Despite these challenges, the use of indices is an important way to communicate and monitor vulnerability, and allows comparisons to be made across geographical areas. Indices can further provide insight into causal processes and exacerbating mechanisms of vulnerability [17]. Through application of robust methods, many pitfalls associated with index construction and communication can be avoided. Several groups have proposed scientific criteria and frameworks to improve the rigour of indicators [47]–[50]. Validation is an additional step to increase reliability of a vulnerability index [51], [52].

### WADI Structure

The WADI is constructed from composite indicators of exposure and susceptibility, where susceptibility represents the existing social, economic or cultural conditions that render a population sensitive to a water-associated pathogen, and exposure represents conditions conducive to the presence and transmission of the pathogen within the environment. The WADI indicators of susceptibility and exposure are comprised of components which are identified using an ‘ecohealth’ conceptual framework, whereby an ‘ecohealth’ approach examines links between humans, the environment and health [53]. The goal of the framework is to facilitate identification of environmental, social and biological factors impacting both susceptibility and exposure to a water-associated disease. Literature review and expert consultation are used to identify these components, which are populated with secondary datasets to construct the WADI. Thus, the WADI can be applied to any water-associated disease by developing a context-specific framework. To establish a proof-of-concept, this paper applied the WADI approach to dengue in Malaysia.

A common challenge in index development is that there is no standard approach to integrate disparate data types which are measured using different metrics, and therefore it is difficult to assign relative importance [22], [54], [55]. The WADI provides a methodology to combine data from biophysical and social environments using a Geographic Information System. The output is a spatially represented index for identification and visualization of areas of vulnerability to a specific water-associated disease. However, because vulnerability cannot be defined solely by the hazard, in context of the WADI disease data is used as a proxy to validate how the index output represents dimensions of vulnerability [56].

A strength of the WADI methodology is its capacity for application in data-poor regions, as global climate, land use and social datasets are available from sources such as FAO GeoNetwork (http://www.fao.org/geonetwork/). However, index development is data driven and requires exploring the most relevant, and available data sets for the highest quality information to populate the indicator components. Thus, the resolution and level of applicability of the overall WADI is defined by the scale of the input data.

## Methods: WADI-Dengue

### Conceptual Framework

**Figure 1:**
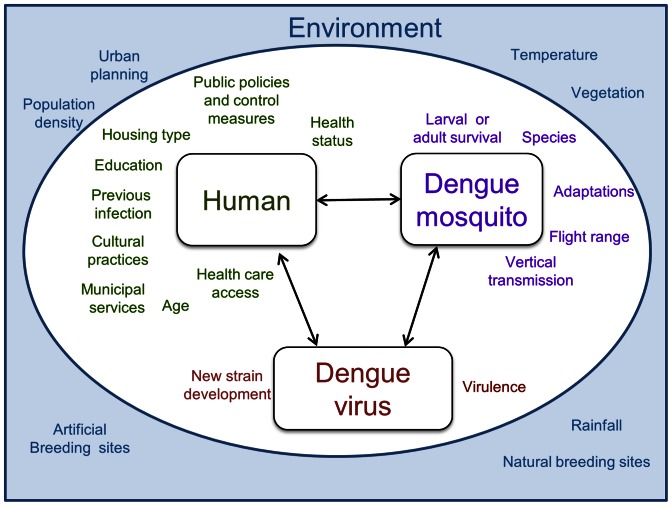
WADI-Dengue conceptual framework describing relationships mediating dengue vulnerability. This framework applies an ecohealth approach which recognizes the inextricable links between humans and their environment, and the ways these influence health.

The first step in applying the WADI tool to dengue involved development of an ecohealth framework to describe linkages between humans, the natural environment, and the dengue mosquito and virus. This framework highlights factors in the social and natural environment that impact vulnerability to dengue by creating conditions of either susceptibility in human communities, or of exposure to the vector and breeding habitats. Drawing on different disciplinary perspectives, elements such as climate conditions, education status and municipal services are incorporated in the framework depicted in Figure 1. While by no means comprehensive, this framework identifies key components that can be used to populate the ‘WADI-Dengue’.

### Data Sources

Datasets used to construct the WADI-Dengue were based on the framework (Figure 1) and the availability of freely accessible data sources, and are listed in Table 1. Because dengue rate data were available at the state level in Malaysia, this dataset was used to identify factors for the index showing a significant association (*p*<0.05). In order to do this a calibration dataset was used consisting of average rates of dengue for odd years from 2001–2011. In the case of similar factors, such as mean and maximum temperature, maximum temperature demonstrated a stronger association and thus this dataset was used for the temperature component. The most up-to-date and complete datasets were used for each component and all states in peninsular Malaysia were included. Dengue rates from even years were used later in the analysis for validation.

**Table 1:** Components of the WADI–Dengue for Malaysia.

Indicator	Component	Dengue WADI factor	Data source
**Exposure**	Climate	Maximum temperature; Precipitation	WorldClim global climate surfaces current conditions [52]
	Land environment	Types of land usse	NUS Centre for Remote Imaging, Sensing and Processing 2010 Southeast Asia Land Cover Map [85]
	Human environment	Population density	Malaysia Census report (2010)
**Susceptibility**	Individual	Age under 15 years	Malaysia Census report (2010)
	Community	Housing quality	Malaysia Ministry of Housing and Local Government (2004)
		Water and sanitation; Health care access	Malaysia Household Income & Basic Amenities Survey report (2009)
		Female Education level	Malaysia Census report (2000)

Monthly climatologies of cumulative precipitation and maximum temperature data were obtained from WorldClim (http://www.worldclim.org/). These consist of global climate grids with a spatial resolution of 1 km^2^ generated by interpolation of average monthly climate data from weather stations [57]. Worldclim uses major climate databases compiled by networks including the Global Historical Climatology Network (GHCN), the FAO, and the WMO.

To prepare the datasets for index construction, component values were assigned a score between 0 and 1, representing a range from low to high exposure or susceptibility. Exposure scores were based on general dengue thresholds identified in the literature, described in the following section and listed in Table 2. Susceptibility scores were created by normalization using the Human Development Index approach described in the index construction section below [43].

**Table 2:** Thresholds used to create the exposure indicator components.

Exposure indicator component	Dimension	Exposure value
**Population density (thousand persons/sq.km)**	<0.10	0
	≥0.10–<0.25	0.25
	≥0.25–<0.5	0.5
	≥0.5–<1.0	0.75
	≥1.0	1
**Land cover component**	Urban	1
	Agricultural/plantation	0.50
	Mixed vegetated/agricultural	0.25
	Forest	0
**Temperature**	Maximum monthly temperature, lag of 2 months	>20°C and ≤34°C : linear increase in exposure up to 1; ≤20°C or >34°C : 0 exposure
**Precipitation**	Monthly cumulative precipitation, lag of 2 months	<300 mm precipitation: linear increase in exposure up to 1; >300 mm monthly precipitation: 0 exposure

### Component Thresholds

Climate conditions, including temperature and precipitation patterns impact the *A. aegypti* mosquito, and have been linked to dengue transmission in many parts of world (eg. [24], [58]–[63]). The effect of temperature and precipitation on dengue is lagged 1–3 months [59], [64], [65], and this is incorporated into the WADI exposure indicator using a 2 month lag. Increasing temperature has a combined effect of impacting virus development as well as vector survival. Longer vector lifespan as well as more rapid viral incubation increases the proportion of infectious vectors [66]. Increased mosquito travel and bite rates also correspond to higher temperature [67], [68]. Dengue incidence is observed to increase linearly with weekly mean temperature, with the greatest relative risk occurring at a time lag of 9–12 weeks [69]. However, models have determined that below temperatures of 20°C and higher than 34°C, *Aedes* mosquito populations cannot reproduce in substantial numbers [68]. Based on these relationships, the exposure temperature component increases linearly from 20°C to 34°C, as described in Table 2.

The incidence of dengue is often higher during wet seasons and increased precipitation has been positively correlated with mosquito reproduction rates and with dengue transmission in many regions [32], [70]–[73]. This is because rainfall fills natural or artificial containers creating mosquito breeding sites. Precipitation does not just create breeding sites; humidity is also linked to *Aedes* fecundity [74]. However, after a certain threshold is reached additional rainfall floods breeding habitats and washes eggs and larvae away [59], [75]. This results in reduced exposure during extremely heavy rainfall events, such as monsoon rains, through the destruction of vector eggs and larvae. Dengue incidence is observed to increase linearly with weekly precipitation at a lag of 5–12 weeks, peaking at 75 mm [69]. Based on these relationships the precipitation exposure component increases to a maximum of 300 mm monthly, as described in Table 2.

#### Population density

Like most water-associated illnesses, exposure to dengue is greater in highly populated areas. Humans are hosts for the virus, and thus the likelihood of transmission increases with increased population density [76]. Correlations between incidence of dengue and population density have been observed in many areas [77]–[79]. In some regions where this relationship was not observed, it was largely explained by high susceptibility due to lack of piped water, such as in rural areas [80], [81]. Population density is classified into five levels between 0 and 1 for the WADI component (Table 2).

#### Landcover

Modification of natural ecosystems by human activities has been associated with emerging and reemerging diseases [82]. *A. aegypti* is an opportunistic breeder, highly adapted to urban and domestic environments [83], [84]. Increased global incidence of dengue has been linked to rapid urbanization for this reason. It breeds in natural as well as artificial sites ranging from water storage containers to defrost trays of refrigerators, pet dishes, waste materials like plastic containers, and flower vases [85], [86]. Meanwhile, higher connectivity of urban areas by transportation networks increases movement of infected individuals, who act as reservoir hosts of the virus. Identifying the type of land favored by vectors in urban and rural areas indicates where humans may be exposed to the dengue virus. Research on habitat gradients for vector species has shown that *A. aegypti* is rarely found in vegetated and forested land, while it dominates high density urban areas [87], [88]. In low density housing in rural areas the secondary vector, *A. albopictus,* predominates, preferring to breed outdoors in vegetated and rural regions [89]. This *Aedes* vector habitat gradient is used to create the landcover WADI component [90], as described in Table 2.

#### Susceptibility components

Components selected to create the susceptibility indicator include demographic and socioeconomic variables at an ecological level. Age, water use, level of female education, access to healthcare and extent of poor quality housing are analyzed in the WADI-Dengue and are listed in Table 3. Age is a susceptibility factor for dengue because children have shown higher sensitivity to severe forms of dengue, such as dengue hemorrhagic fever and dengue shock syndrome [91]–[93]. The risk of death from DHF is much higher in children compared with adults, possibly due to capillary fragility in children [94], [95]. Dengue is a major cause of hospitalization and death of children in some Asian and Latin American countries [3], [84]. School-aged children are especially vulnerable to infection because school buses and school yards present opportunities for human-vector-human transmission during early morning biting hours [96], [97].

**Table 3:** Susceptibility components and their dimensions.

	Component	Dimension
Individual	Age under 15 years	% population under 15 years by state
Community	Housing quality	Number of households living in squatter settlements by state
	Water and sanitation	% Households using pour flush toilets by state
	Health care access	% Households >5 km from health clinic by state
	Female education level	% Females **not** completing at least some secondary schooling by district

Water use and behavior is an important determinant of susceptibility to water-associated disease. Improving domestic water supplies used for drinking, sanitation and hygiene is important for reducing vector populations because the use of indoor and outdoor water storage containers creates potential breeding sites [98]. In regions without a reliable piped water supply, storing water for purposes of drinking or for using pour flush toilets increases susceptibility (eg., [76], [80], [99], [100]). Even where piped water is available, pour flush toilets are used in many rural areas which often entail containers of stored water in the bathroom [101].

Poor housing quality, such as that found in slums, squatter settlements and rapidly expanding peri-urban regions increases susceptibility to dengue [89]. Lack of window and door screens, which is common in underprivileged areas, allows free passage of mosquitoes between the interior and exterior [84], [102]. Conversely, use of air conditioners and sealed windows and doors reduce susceptibility [103]. Housing with porous floors, unplastered walls, or untiled bathrooms can increase humidity indoors and is conducive to vector survival [104]. Additionally, temporary houses are often associated with a lack of other infrastructure and services, such as adequate waste disposal; dump sites can collect water and provide breeding sites for mosquitoes.

Low education level is a commonly used indicator of susceptibility, as in the Human Development Index [43]. Increases in adaptive capacity are observed in families with increased female education and literacy [105]. Adaptive capacity is the ability or potential to cope with a hazard and to reduce the likelihood of harmful effects [106]. This is especially important in the case of water-associated disease, as females in many countries are responsible for domestic water-related tasks such as water collection and storage and food preparation [107]. Being able to read and understand public health messages regarding dengue prevention and early recognition of symptoms enhances resilience to the disease. Within this context, completion of primary education is a key threshold, as highlighted by the Millennium Development Goal for universal primary education [108]. Women who have received primary education are associated with increased health outcomes, and in poor households this education offers a protective effect [109], [110]. To capture this, the WADI uses the proportion of females who have completed at least some secondary schooling to calculate this component.

Adequate access to healthcare creates resilience to water-associated disease, and reduces dengue fatality significantly [3], [84]. However, differences in access to health care services exist within a country or region due to a range of barriers or facilitators [111]. In the case of dengue in infants and children, hospitalization and deaths in hospital have been associated with delays in presentation for medical attention, diagnosis and appropriate care [112]. Dengue, as well as many other infections such as malaria and measles, generally presents with fever, which can cause confusion over diagnosis and severity of the illness, and result in delayed treatment [113].

### Index Construction

Datasets for components were imported into the geographical information system (GIS) ArcGIS version 10, and converted into raster format for manipulation (ESRI, Redlands, CA). Exposure component raster layers, based on the thresholds described above and listed in Table 2, contained pixels representing a value from 0 to 1. Temperature and precipitation rasters were developed for each month, resulting in 12 temperature and 12 precipitation layers. Susceptibility component raster layers also contained pixels representing a value from 0 to 1, and were created by normalization of component data using the Human Development Index approach, where x represents the factor in question, and x_min_ and x_max_ represent the lowest and highest value in the dataset respectively:



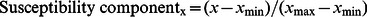
(1)A detailed investigation of the relative contribution of each individual component within the exposure or susceptibility indicators was not possible due to data limitations, so the components were assumed to have equal impact and were aggregated to form a composite indicator using an arithmetic average [55]. In the WADI-Dengue example each component was weighted equally within the indicator; however these can be weighted differently in other disease applications of the WADI.

A weighting of exposure and susceptibility indicators to construct the final index was based on contribution to overall vulnerability. Weightings of exposure and susceptibility indicators were tested to determine the optimal contribution of each to the WADI-Dengue. The weighting scheme with the strongest association between the WADI-Dengue and the calibration dataset (average rates of dengue for odd years from 2001–2011) was identified based on the highest Pearson’s correlation coefficient and used to create the final index. Depending on the transmission pathways associated with a water-associated disease, weightings of the exposure and susceptibility components used in the WADI may change [10].

The final stage of the methodology involved map creation using the GIS layers produced in the index construction. While many vulnerability assessment approaches provide tabular outputs, visualization is an important part of the WADI methodology because it allows users to gain a better grasp of the spatial distribution of regions of high or low vulnerability. End-users, and especially those without a medical background, can easily see and interpret the index output in this format compared to other formats [114].

### Accessibility

The raster manipulation approach was successfully tested in an open source GIS software package GRASS, which is specialized in raster processing (http://grass.osgeo.org/). This means that users are able to construct and visualize the WADI in contexts where proprietary GIS packages are not available.

### Validation

While many vulnerability assessment approaches apply rigorous methodological steps to generate an index there are often barriers to validating findings [51]. Because vulnerability is a holistic concept incorporating complex interactions between social and biophysical dimensions, it is difficult to find empirical evidence for validation and it is often indirectly measured [115]. In cases where an independent dataset is available as a proxy for vulnerability, statistical approaches can be used to validate the methodology. However, while statistical validity may be demonstrated, conceptual validity is another consideration as the theoretical importance of certain components may be different from the statistical importance [50], [51]. Despite constraints associated with validating vulnerability assessments, rates of dengue were used to demonstrate how the WADI-Dengue represents vulnerability within Malaysia. Correlation analysis was used to examine the relationship between the average WADI-Dengue values per state for each month of the year and a validation data series, consisting of monthly state-level dengue rates for even years over the period 2001–2011. A test for autocorrelation was conducted due to the temporal dimension of the index. Finally, consultations with vector control staff in Malaysia were conducted to discuss the map outputs within the context of the current dengue situation. This triangulation approach provides an additional perspective in the validation process [17], [116].

## Results

Weighting schemes were tested to determine the contribution of exposure and susceptibility composite indicators to the WADI-Dengue. A weighting of 1 for susceptibility and 3 for exposure indicated the largest correlation coefficient (r) of 0.71 (*p*<.0001). These weightings were used to create the WADI-Dengue and map outputs.

**Figure 2:**
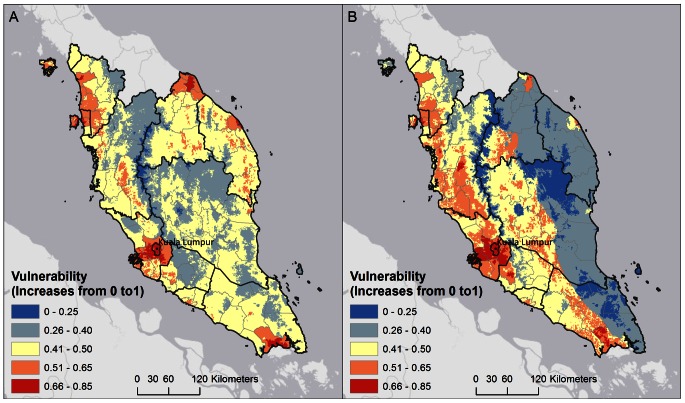
WADI output for June (A) and December (B). This visualization of the WADI output for June (A) and December (B) shows vulnerability increasing from 0 towards 1. Areas with both high exposure and susceptibility resulted in the highest overall vulnerability, and represent priority areas for intervention planning. Urban environments, especially the Kuala Lumpur region, are highlighted as highly vulnerable areas. The comparison between June and December WADI output illustrates the change in vulnerability over the year due to the temporal changes in climate patterns. Eastern Malaysia is strongly affected by the monsoon season, which brings heavy rainfall to the coast for several months at the end of each year, possibly washing away mosquito breeding sites. However, in the drier months exposure in this region increases possibly due to more moderate rainfall conducive to larger mosquito populations, resulting in higher vulnerability. In contrast, some areas of Malaysia have consistently low vulnerability due to low exposure from forested areas which are not favorable for *Aedes* vectors, as well as low population density. Zero vulnerability is observed in areas where temperatures are too low to be conducive to mosquito survival, which is confined to mountainous regions in central Malaysia.

Map outputs of the WADI-Dengue in Malaysia indicate several significant patterns. Based on the WADI approach, areas with both high exposure and susceptibility have the highest overall vulnerability, and represent priority areas for disease control and health promotion planning. In Malaysia, high vulnerability is observed throughout the year in urban areas, especially in the capital Kuala Lumpur and the surrounding region. However in other regions vulnerability varies throughout the year due to the influence of changing climate patterns. These trends indicate that several key drivers are operating and contributing to overall vulnerability. In Kuala Lumpur, while the exposure indicator is high and carries a greater weighting, the susceptibility indicator is low due to higher adaptive capacity in this region. This adaptive capacity stems from factors such as high female education levels, low use of pour-flush toilets and higher access to healthcare. In some regions where exposure is moderate or low, such as the east coast of peninsular Malaysia, vulnerability increases due to higher susceptibility, such as lower access to healthcare, and higher use of pour-flush toilets which require storage of water that creates potential breeding sites. This region is strongly affected by the monsoon season, which brings heavy rainfall to the eastern coast for several months at the end of each year. This rainfall can wash away mosquito breeding sites and the exposure indicator is low during these conditions. In the drier months, exposure in this coastal region increases due to more moderate rainfall which is thought to be conducive to larger mosquito populations. In combination with high susceptibility, this creates higher overall vulnerability during drier conditions. In contrast, western Malaysia is sheltered from heavy monsoon rains by a central mountainous region. Figure 2 shows vulnerability outputs for June and December, which illustrate these changes in vulnerability over the year due to the temporal changes in climate patterns. In contrast, some areas of Malaysia have low vulnerability due to low exposure from vegetated and forested areas which are not favourable for *Aedes* vectors, and low population density which reduces available dengue virus reservoir hosts. Zero vulnerability is observed in areas where temperatures are too low to be conducive to vector survival, confined to mountainous regions in central Malaysia.

### Validation

**Figure 3:**
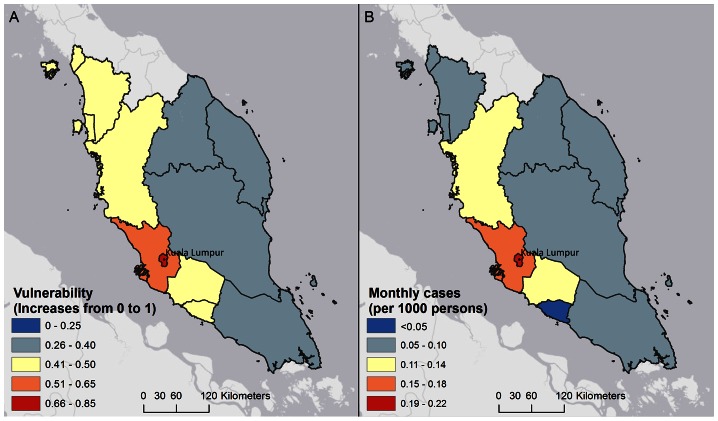
Comparison of vulnerability at state level in December (A) and dengue rates by state in February (B). These maps compare vulnerability averaged at the state-level (A) with dengue rates per 1000 persons (B), in Malaysia. Average dengue rates during February for even years from 2001 to 2011 are shown as this dataset was used to validate the WADI-Dengue case study. Dengue rates are shown for February because the WADI-Dengue uses a 2 month lag for climate data, based on the exposure thresholds identified in the methods section. Although averaged vulnerability values hide features such as urban areas and mountainous regions, the lower rates of cases observed on the east coast of Malaysia during the monsoon season (November – January) are consistent with the vulnerability profile.

The WADI-Dengue values by state were found to be significantly associated with the validation dataset consisting of state-level monthly dengue rates for even years from 2001 to 2011, (correlation coefficient of 0.71, *p*<.0001). Results of a linear regression analysis are shown in Table 4 (R^2^ = 0.50). Due to the temporal nature of the data, autocorrelation among regression residuals was examined using the Durbin–Watson test [117]. Figure 3 shows average dengue rates in February using the validation dataset, compared with the vulnerability output for December averaged at state-level. Dengue rates are shown for February because the WADI-Dengue uses a 2 month lag for climate data, based on the exposure thresholds identified in the methods section. As discussed above, low vulnerability is observed in eastern Malaysia due in part to heavy rainfall that occurs during this season. In addition to the statistical validation, results from key informant consultation with national vector control staff in Malaysia supported many of the patterns observed in the map outputs, including the high burden of illness in urban areas and the seasonal variability in eastern Malaysia. More specifically, the eastern Malaysia region was recognized by key informants to suffer from higher dengue rates during drier months from June to September, a pattern that clearly emerged from the WADI-Dengue map outputs.

**Table 4:** Regression results from the validation of the WADI-Dengue.

Coefficient	P value	95% CI
0.63	<.0001	0.53–0.74

## Discussion

Water-associated diseases without vaccines or cures, such as dengue, require integrated approaches that reduce vector or pathogen exposure as well as human susceptibility to the disease. The objective of this study was to develop the WADI tool as an evidence-based approach to mapping vulnerability, using dengue as the water associated disease of interest. The tool can help users to think critically about water-associated disease transmission processes and bring attention to priority areas to focus interventions, such as through evaluating the need for increased vector control resources in areas with high exposure or implementing education programs to increase adaptive capacity in areas with high susceptibility.

The WADI-Dengue was applied and validated at a sub-national level in Malaysia, illustrating differential patterns of vulnerability based on climate trends and social determinants at a macro scale. While the holistic nature of the WADI instrument presents methodological challenges associated with using data measured with different metrics, a major advantage of the tool is the potential to use the rich array of available datasets and models to populate the index.

In this WADI-Dengue example, the weighting approach was balanced more heavily on exposure than susceptibility. This could indicate that social drivers operate at a smaller scale than the state-level, a data constraint resulting from the highest resolution some data were freely available. These state level data may hide variability in smaller regions, and higher resolution information on susceptibility may enhance the contribution from this indicator to the WADI-Dengue. Furthermore, factors not available for the WADI-Dengue application may be important, such as activity of community dengue programs, extent of media campaigns and differential funding for dengue control programs. While capacity to adapt and cope with a health hazard is an element of vulnerability, this was not directly assessed as an indicator in the WADI-Dengue case study. However a high score in some WADI susceptibility components such as female education level suggests a lower coping capacity.

In addition, the results emphasize the importance of exposure factors in the context of vulnerability to dengue in Malaysia, as well as the need for additional work to examine how dynamic processes such as monsoon rains impact dengue transmission in this region. Although the statistical validation used in this case study is only one approach to index validation, it should be emphasized that theoretical validation of the WADI-Dengue is provided by both the conceptual framework and component thresholds developed for the index.

A limitation highlighted by the WADI-Dengue applied in Malaysia is the poor availability of disease estimates in many low or middle income regions. In Malaysia, the availability of dengue case records at a state-level limits the validation approach to that level, providing information on large-scale patterns in vulnerability and not on processes occurring at small scales. There is a push for better global health data for evidence-based decision-making, which has included water-associated diseases such as dengue with projects to improve dengue distribution maps [118] and inclusion of dengue in the most recent publication of the Global Burden of Disease Study [119]. Improved capacity to collect estimates of water-associated disease, especially at sub-national levels, would strengthen the validation process and implementation of the WADI tool.

A lack of tools and data resources for vulnerability assessment and mapping has been identified as a key gap in the field [56], [120]. This is especially true at the water-health nexus, as health impacts are primarily examined through risk models which calculate the probability of disease transmission. Predictions from early warning systems provide critical information for immediate on-the-ground actions such as insecticide fogging, quarantines and media releases. However in many contexts, predictive models are limited because the use of early warning systems relies on large inputs of financial, human, and data resources and an adequate public health infrastructure [28]. While the WADI tool is informative rather than predictive, the output describes differential conditions of vulnerability that can provide key information for decision-makers planning longer term interventions.

The WADI is a fast and inexpensive process compared with collecting primary data; although using secondary data can be limiting when ideal component datasets identified in the framework are difficult to obtain or non-existent. In addition, this pragmatic tool can be applied and refined to a smaller scale context such as a region of interest within a country by using spatially higher resolution datasets as well as environmental data across a specific time frame. For example, where meteorological records are collected these can be used instead of climatalogies like the WorldClim dataset. However, using these more local datasets often requires interpolation of weather station records, a capacity that may not always be available to public health practitioners who want to identify areas of vulnerability. Finally, the WADI vulnerability mapping approach is flexible and can be tailored to different water-associated disease contexts by identifying specific social and biophysical determinants that influence exposure, susceptibility and ability to cope within a range of environments.

### Conclusions

The WADI is a holistic tool for assessing vulnerability to water-associated disease based on differential conditions of exposure, susceptibility and capacity to cope. This case study of the WADI tool, developed for dengue in Malaysia, showed applicability and validity of the approach on a large scale. It demonstrated that uniform disease intervention strategies may not be appropriate, even within a country, as the roots of vulnerability are different across geographical areas. Like many index approaches, the WADI is limited by the way complex information is reduced to a simple output and the availability of detailed subnational health data for validation purposes. However this tool has important policy implications; an aggregated value is helpful for decision-makers who must otherwise draw individual conclusions about many different elements mediating transmission of water-associated diseases.

The more comprehensive understanding provided by a vulnerability assessment can be a significant contribution to the development of downstream initiatives, such as early warning and surveillance systems. To increase user confidence in the applicability of the approach, future work will examine the sensitivity of the WADI tool to changes in its construction, the scale at which the WADI is applied and its use in different geographic contexts [121].

As global environmental change, including climate change, is expected to increase pressure on disease transmission processes, integrated tools such as the WADI will become more critical. The WADI methodology can be extended with the use of scenarios or projected data (e.g. climate change, land use change, or population density projections) to better understand the dynamic nature of vulnerability to water-associated disease. For instance, some areas in Malaysia such as the highlands could suffer expansion of vulnerable areas if temperatures increase enough to support dengue vector populations, a future application of the tool. The approach also has potential to be incorporated with other types of vulnerability assessment, such as for floods or droughts, and forms part of an emerging suite of tools available for vulnerability assessment at the water-health nexus.
